# T Cell Defects and Immunotherapy in Chronic Lymphocytic Leukemia

**DOI:** 10.3390/cancers13133255

**Published:** 2021-06-29

**Authors:** Elisavet Vlachonikola, Kostas Stamatopoulos, Anastasia Chatzidimitriou

**Affiliations:** 1 Centre for Research and Technology Hellas, Institute of Applied Biosciences, 57001 Thessaloniki, Greece; e.vlachonikola@certh.gr (E.V.); kostas.stamatopoulos@certh.gr (K.S.); 2 Department of Genetics and Molecular Biology, Faculty of Biology, Aristotle University of Thessaloniki, 54124 Thessaloniki, Greece; 3 Department of Molecular Medicine and Surgery, Karolinska Institutet, 17177 Stockholm, Sweden

**Keywords:** chronic lymphocytic leukemia (CLL), tumor microenvironment (TME), T cells, immunotherapy

## Abstract

**Simple Summary:**

The treatment of chronic lymphocytic leukemia (CLL) is a rapidly evolving field; however, despite recent progress, CLL remains incurable. Different types of immunotherapy have emerged as therapeutic options for CLL, aiming to boost anti-tumor immune responses; that said, despite initial promising results, not all patients benefit from immunotherapy. Relevant to this, the tumor microenvironment (TME) in CLL has been proposed to suppress effective immune responses while also promoting tumor growth, hence impacting on the response to immunotherapy as well. T cells, in particular, are severely dysfunctional in CLL and cannot mount effective immune responses against the malignant cells. However, reinvigoration of their effector function is still a possibility under the proper manipulation and has been associated with tumor regression. In this contribution, we examine the current immunotherapeutic options for CLL in relation to well-characterized T cell defects, focusing on possible counteracts that enhance anti-tumor immunity through the available treatment modalities.

**Abstract:**

In the past few years, independent studies have highlighted the relevance of the tumor microenvironment (TME) in cancer, revealing a great variety of TME-related predictive markers, as well as identifying novel therapeutic targets in the TME. Cancer immunotherapy targets different components of the immune system and the TME at large in order to reinforce effector mechanisms or relieve inhibitory and suppressive signaling. Currently, it constitutes a clinically validated treatment for many cancers, including chronic lymphocytic leukemia (CLL), an incurable malignancy of mature B lymphocytes with great dependency on microenvironmental signals. Although immunotherapy represents a promising therapeutic option with encouraging results in CLL, the dysfunctional T cell compartment remains a major obstacle in such approaches. In the scope of this review, we outline the current immunotherapeutic treatment options in CLL in the light of recent immunogenetic and functional evidence of T cell impairment. We also highlight possible approaches for overcoming T cell defects and invigorating potent anti-tumor immune responses that would enhance the efficacy of immunotherapy.

## 1. Introduction

Chronic lymphocytic leukemia (CLL) is a malignancy of mature, antigen-experienced B cells with a distinct immunophenotype (CD5^+^CD23^+^sIg^low^) [1]. CLL affects mainly aged individuals and is characterized by a highly variable clinical course, extending from asymptomatic to refractory/progressive disease. Despite remarkable recent progress in the management of CLL, a definitive cure is still an unmet need [2]. Ample evidence suggests that this clinical heterogeneity is linked to and probably reflects the underlying biological heterogeneity [2,3,4,5].

Molecular cues support that the unique characteristics of the clonotypic B cell receptor immunoglobulin (BcR IG) strongly affect the intensity of downstream intracellular signaling cascades in CLL cells, regulating their proliferation and survival, and, eventually, also impacting on the clinical course. At the genetic level, a great variety of genetic lesions, both copy number variations (deletions of chromosomes 13q, 17p, 11q and trisomy 12) and recurrent gene mutations (*TP53*, *SF3B1*, *MYD88*, *NOTCH1*, and *ATM*), have been identified with variable frequency [2,6]. Although evidence of a sole driver lesion is lacking in the pathophysiology of CLL, the prognostic relevance of these aberrations is well established, with subgroups defined by a particular aberration displaying distinct clinical course and outcome [7,8]. Adding to this complex picture, epigenetic mechanisms seem to also play a role in the natural history of CLL, with significant differences in methylation signatures reported for CLL cells versus normal B cells, as well as between different patients [2,9,10,11].

Along with the complex (epi)genomic landscape of CLL, the TME plays a crucial role in CLL cell dynamics. The great majority of circulating CLL cells are inactive; however, there is a small proliferative fraction of CLL cells that homes into the proliferation centers of the bone marrow (BM) and secondary lymphoid organs [12,13]. These anatomical sites represent the theatre of continuous bi-directional interactions between CLL cells and components of the TME [14]. The cellular compartment of the CLL TME is composed of different cell populations, including T cells, mesenchymal stromal cells and monocyte-derived nurse-like cells (NLCs), which secrete a great variety of soluble molecules, mostly chemokines, that control cell trafficking [15].

Early studies evinced the dependency of CLL cells on the TME, as these cells underwent spontaneous apoptosis in cell cultures [16], whereas they were partially rescued only in co-cultures with BM stromal cells (BMSCs) and NLCs [17,18]. Accumulating evidence supports that microenvironmental signals also drive disease progression through the suppression of immune responses [19].

This concept of a supportive milieu that promotes leukemogenesis has revolutionized our knowledge on the mechanisms supporting CLL cell survival and expansion, whereas it has highlighted new therapeutic targets for CLL regression.

## 2. The Tumor Microenvironment in Chronic Lymphocytic Leukemia: Supportive and Suppressive

The main characteristic of the CLL TME concerns the reciprocal crosstalk between the malignant cells and the various bystander cells that leads to dynamic modifications in both parties. Although the exact mechanism remains poorly understood, the surrounding microenvironmental cells, such as T cells, dendritic cells, macrophages and mesenchymal stromal cells, appear remodeled in order to provide trophic signals to the CLL cells, promoting resistance to apoptosis and homing to lymph nodes [20]. Importantly, CLL cells can actively reinforce supportive functions of the cells in the TME either by direct cell contact or through indirect communication based on soluble factors and extracellular vesicles [21,22,23].

CLL cells are also able to interfere with and perturb anti-tumor immune responses [24]. The immunomodulatory capacity of CLL cells mostly relies on the expression of a great variety of inhibitory molecules on their surface that restrain the effector arms of the immune system [25]. Of note, the main target of the CLL suppressive mechanisms is the T cell compartment; however, the influence of T cells on disease evolution remains contested [14].

## 3. Immunotherapy in CLL

Immunotherapy has revolutionized cancer treatment, achieving promising results in terms of durable responses and overall survival (OS) in cancer patients. Although the TME in cancer is polarized towards a supportive niche that promotes tumor growth, the immune system holds the ability to mount strong anti-tumor responses under the proper activation, and this is key to immunotherapy. Of note, the success of immunotherapy is directly related to the specific features of the TME; therefore, studies elucidating the mechanisms that drive immune suppression and promote tumor growth remain of great relevance [26].

Intense research over the past few years on the features and interactions within the CLL TME disclosed a plethora of new targets for therapeutic manipulations, while also providing the rationale for novel immunotherapeutic approaches. Immunotherapy in CLL can be broadly subdivided into two different categories: (i) passive immunotherapy based on the use of monoclonal antibodies and immunomodulating agents, and (ii) active immunotherapy, referring to vaccines and adoptive cell transfer models.

In the following paragraphs, we will attempt a concise overview of both passive and active immunotherapy in CLL. This overview is not meant to be exhaustive (indicatively, only anti-CD20 monoclonal antibodies are covered) but rather aims to illustrate concepts and mechanisms and highlight the potential of immunotherapy for the improved management of patients with CLL.

### 3.1. Passive Immunotherapy in CLL

#### 3.1.1. Anti-CD20 Monoclonal Antibodies

Monoclonal antibodies against various antigens expressed on the surface of the malignant cells represent a successful immunotherapeutic modality in CLL. In the 2000s, the addition of the anti-CD20 monoclonal antibody rituximab to chemotherapy led to the establishment of Fludarabine-Cyclophosphamide-Rituximab (FCR) as a highly effective regimen for fit and previously untreated CLL patients [27]. For many years, FCR has outperformed any previous regimen in terms of overall response (OR); moreover, long-term follow-up of treated patients demonstrated durable remissions leading eventually to increased OS [27,28]. Rituximab targets the CD20 transmembrane receptor [29], a ubiquitous phenotypic marker of CLL cells and, for that matter, all mature B cells. Rituximab primes CLL cells for complement-mediated lysis and antibody-dependent cell-mediated cytotoxicity, whereas it seems to interfere in signaling pathways and Ca2^+^ flux within the malignant cells [30,31]. Ofatumumab and Obinutuzumab, novel generation anti-CD20 monoclonal antibodies with similar albeit distinct mechanisms of action, have also been used in combination with mostly chlorambucil for “unfit” patients, leading to significant improvements in outcome [32,33,34,35].

Overall, the advent of anti-CD20 monoclonal antibodies constituted a paradigm shift in the treatment of CLL. That said, the emergence of novel agents has led to yet another paradigm change towards the chemotherapy-free treatment of CLL with greater efficacy and less toxicity [36]. Anti-CD20 monoclonal antibodies are often combined with novel agents and regulatory approvals for such regimens have already been granted; admittedly, however, their precise role in this new setting remains to be fully elucidated [37,38,39].

#### 3.1.2. Immune-Checkpoint Inhibitors

A great variety of antibodies under the name of “immune-checkpoint inhibitors” (ICIs) target the inhibitory axis that emanates from CLL cells towards bystander cells, particularly T cells, through the overexpression of inhibitory molecules on their surface.

One of the best characterized mechanisms through which CLL cells suppress T cell immune responses is through the overexpression of programmed cell death ligand 1 (PD-L1). PD-L1 binding with the respective PD-1 receptor on T cells leads to CD8^+^ cell silencing and the establishment of general T cell exhaustion, a hallmark of CLL [40,41,42]. Pharmacological manipulation of the PD-1/PD-L1 axis with the use of anti-PD-L1 monoclonal antibodies (nivolumab and pembrolizumab) has already demonstrated positive results in murine models of CLL, inverting the pro-tumoral state of T cells [42], similar to what has been reported in solid cancers as well [42,43]. Pembrolizumab has documented clinical efficacy in CLL patients with Richter’s transformation [44] and nivolumab is active, albeit only modestly, in relapsed/refractory CLL [45]. Moreover, evidence from murine models further suggests that combinatorial protocols of ICIs with signaling inhibitors (particularly B cell receptor inhibitors—BCRi) hold promise for the future [44,46,47].

#### 3.1.3. Bispecific T Cell Engager Antibodies

Bispecific T cell engager antibodies (BiTEs) are binary antibodies that on the one hand engage on T cells and on the other target a tumor-specific antigen. In that way, T cell effector functions and cytotoxicity are triggered and driven towards the malignant cells, in a process resembling the natural mechanism of immune synapse formation [48,49,50]. Blinatumomab, a CD3/CD19 BiTE, is the first approved regiment for clinical use in this category after the impressive results demonstrated in patients with relapsed/refractory B acute lymphoblastic leukemia (B-ALL) [48,49].

In vitro studies in CLL patient samples indicated that blinatumomab could reverse the exhaustion state of T cells, as it could induce T cell activation and proliferation. More particularly, the conjugation of T cells with CLL cells triggered cytokine secretion and granzyme B release that led to increased cytotoxicity, similar to what is occurring during normal immune responses [51]. Furthermore, this BiTE increases the functional competence of T cells in cultures with primary cells derived from CLL patients previously treated with the BCRi ibrutinib, suggesting novel powerful immunotherapeutic strategies in CLL [52].

#### 3.1.4. Immunomodulatory Drugs

Immunomodulatory drugs (IMiDs) represent a different category of immunotherapeutic agents for the treatment of CLL, particularly for the relapsed/refractory setting [53]. Lenalidomide, a thalidomide-analog approved for treatment on multiple myeloma, myelodysplastic syndromes and mantle cell lymphoma, is the most used IMiD in CLL [54,55,56]. Encouraging results from single-agent trials proposed lenalidomide as a valid option also for first-line treatment in untreated and/or elderly patients with CLL, with an overall response rate ranging from 56 to 65% [57,58]. Combinations of lenalidomide with anti-CD20 monoclonal antibodies (rituximab or ofatumumab) have been tried in patients with relapsed/refractory disease, displaying superior overall response rates, albeit with increased toxicity [59,60,61].

Although its exact mechanism of action is not yet fully elucidated, lenalidomide reverses some of the established immune defects in CLL patients, also surpassing the immune tolerance by modulation of several different cell populations, including B and T cell along with NK and dendritic cells [54]. In more detail, lenalidomide administration appears to inhibit pro-survival signaling through reducing the expression of different cytokines, such as TNF-α and IL-6 [53]. The multifaced action of lenalidomide targets different T cell subpopulations, leading to the restoration of effector functions and the enhancement of CD8^+^ cell-mediated cytotoxicity, while also controlling the number and functions of suppressive populations, i.e., T regulatory (Tregs) and Th17 helper cells [54,55]. In parallel, lenalidomide modulates the expression of different surface molecules on CLL cells, affecting their adhesion and cell migration properties and, indirectly, promoting CLL cell apoptosis [54].

Importantly, another potential mechanism of action for lenalidomide concerns the inhibition of angiogenic responses. Extensive angiogenesis in CLL patients has been linked to disease severity and is likely driven by high levels of major angiogenic factors, such as VEGF, expressed by the malignant clone or bystander cells [62,63]. In addition, CLL cells also express the VEGF receptors (VEGFR-1 and 2), hence maintaining an autocrine signaling loop that substantially contributes to tumor progression [63,64]. The interference of angiogenic processes by lenalidomide also impacts on the T cell compartment, as increased expression on VEGF has been linked to T cell immunosuppression through the presence of elevated numbers of Tregs in the TME and/or the direct suppression of T cell proliferation and cytotoxicity (effector T cells express VEGFR-2) [65].

### 3.2. Active Immunotherapy

Although cytotoxic immune responses against malignant cells indeed occur in cancer patients, in most instances effective tumor regression cannot be achieved. The identification of antigens deriving from overexpressed molecules or mutated genes in malignant cells has highlighted new possible targets for anti-tumor immune responses orchestrated by T cells [66]. However, the reduced immunogenicity of these antigens by several modifications through the mechanism of immunoediting, along with tumor-induced immunosuppression and T cell defects, leads progressively to immune silencing, also driving malignant cells to evade immune surveillance [67]. New approaches based on cancer vaccines and modified anti-tumor T cells that invigorate the host immune responses are currently an appealing alternative for immunotherapy and will be presented below. 

#### 3.2.1. Vaccines

Cancer vaccines boost immune responses against malignant cells, contributing to tumor regression. The main mechanism to achieve that is through the stimulation of tumor-reactive cytotoxic T lymphocytes (CTLs). Further activation and proliferation of the CTLs will drive their expansion and unleash anti-tumor responses, whereas the establishment of immunological memory will also provide long-term anti-tumor immunity [68].

One of the most widely used cancer vaccination approaches regards peptide vaccines. The peptides used in cancer vaccine design derive mostly from two broad categories of antigens: (i) tumor-associated antigens (TAAs) and (ii) tumor-specific antigens (TSAs) [69]. The former category is characterized by low tumor-specificity and consists of mainly overexpressed antigens on the malignant cells or antigens that are expressed upon malignant transformation, while the latter category includes high specificity antigens on malignant cells derived from genomic aberrations (e.g., mutations and chromosomal translocations) [70,71]. Nevertheless, the identification of such antigens to use for vaccination is not always straightforward. Other vaccination strategies, including dendritic cell vaccines to induce antigen-presentation and tumor-cell vaccines of either whole cancer cells or genetically modified cells have emerged [59,72]. Although preclinical studies have demonstrated promising results, this has not been translated into clinical efficacy mostly due to the complex mechanisms that regulate immune responses in the TME (e.g., inhibitory or co-stimulatory signals); moreover, the tumor burden also appears to be a major determinant of success [73].

Although CLL is characterized by a great variety of recurrent mutations and genomic aberrations, the identification of highly immunogenic epitopes remains elusive. Consequently, numerous alternative vaccination approaches have been examined, mostly based on the usage of autologous modified CLL cells that express different cytokines or co-stimulatory molecules [73,74]. Unfortunately, despite positive outcomes in preclinical studies, translation into the clinical setting was undermined by the inclusion in these trials of heavily pretreated relapsed/refractory patients; moreover, the compromised functions of T cells possibly contributed to the dampening of effective immune responses [75,76]. However, in view of new evidence regarding the identification of TAAs and the unique properties of the CLL TME, vaccination in CLL still holds promise for the future, mostly in combination with other immunotherapeutic agents [69,77].

#### 3.2.2. CAR-T Cells

Adoptive cell transfer (ACT) refers to the usage of anti-tumor T lymphocytes (autologous or allogenic) that have expanded ex vivo as a driver of tumor regression [78]. The main concept relies on the reintroduction into the immune system of activated anti-tumor T cells that will surpass the inherent dysfunctions and supportive TME and attack malignant cells, while at the same time eliciting immune memory responses for long-lasting remission. The first successful attempt of ACT concerned the administration of tumor-infiltrating lymphocytes (TILs) after lymphodepletion in patients with metastatic melanoma [79]. A more recent approach involves genetic modifications that make T cells express a particular T cell receptor (TR) that has emerged by the recognition of a specific TAA (intra- and/or extracellular), as well as the synthetic construction of chimeric antigen receptors (CARs) that bind on antigens on the surface of the malignant cells [80]. CAR-T cells have attracted most interest and are widely used as a therapeutic option in B-cell malignancies [81].

The generation of CAR-T cells includes the isolation of a patient’s T cells, ex vivo genetic modification in order to express an antigen-specific TR and expansion in culture before the reinjection of these engineered and activated lymphocytes into the patient [81]. The CAR has the advantage of targeting tumor cells in an HLA-independent manner and is structurally composed of a single-chain variable fragment (scFv), resembling a fragment antibody which is intracellularly linked with TR signaling domains [82]. Aiming to increase CAR’s efficacy and expansion, new intracellular domains are constantly added to the main CAR in order for multiple costimulatory domains to be mediated, resulting in different generations of CARs. The most widely used CAR-T system in B-cell malignancies is the CD19-targeted CAR T cells which are already approved for the treatment of pediatric B-ALL and diffuse large B-cell lymphoma after impressive results in clinical trials [83,84].

In CLL, CARs failed to gain ground mostly due to inconclusive results likely as a result of heterogeneous study groups in the trials; in fact, most CLL patients that received CAR-T cells were heavily pre-treated and with poor prognosis [85]. Moreover, the intrinsic characteristics of the TME in CLL, namely, immunosuppression and T cell exhaustion driven by the malignant clone, possibly contribute to the reduced efficacy of CAR-T cells. Allogeneic CAR-T cells from healthy donors have been proposed as an alternative, whereas combination with ibrutinib has also been examined with positive results [85,86].

### 3.3. Immunomodulation as an Off-Target Effect of B Cell Receptor Inhibitors

Targeting the proliferative fraction of CLL cells through the inhibition of the signals transmitted by the BcR proved to be a successful therapeutic strategy with remarkable results in both treatment-naïve patients and relapsed/refractory CLL cases [87]. The most prominent compounds in this category are ibrutinib, an inhibitor of the Bruton’s tyrosine kinase (BTK), and idelalisib, a selective inhibitor of the lipid kinase PI3Kδ [88]. Besides effectively targeting signaling pathways in CLL cells, these agents have off-target effects in bystander T cells due to cross-reactivity on functionally similar kinases of the T cells, leading to dynamic changes of their immune properties and functions.

Indeed, inhibition of ITK (a member of the same family as BTK, and also structurally and functionally similar to BTK) in T cells by ibrutinib resulted in the expansion of functionally competent T clones with improved capacity for effective immune synapse formation and polarization towards Th1 immune responses [89,90,91]. On this evidence, combination protocols of ibrutinib with ICIs, CAR-T cells and BiTEs are already being evaluated in clinical rials, with preliminary evidence suggesting the combinations’ superiority versus the respective monotherapies [47,92,93,94,95,96].

Thus, although not formally classified as immunotherapeutic agents, off-target effects of the BCRis have highlighted them as promising novel agents for combinatorial protocols targeting not only the malignant CLL cells but also the CLL TME. Nevertheless, great caution is warranted regarding the potential severe adverse effects from such approaches manifesting as autoimmune complications or, rarely, cytokine release syndrome [87,97].

## 4. Why Does Immunotherapy Work for Some but Not All Patients?

An improved understanding of TME-related mechanisms mediating CLL cell survival and proliferation has led to significant therapeutic advances, even in adverse-prognostic cases. That said, real-world evidence indicates that when it comes to active immunotherapy, only a fraction of patients with CLL will benefit and even less will experience durable remissions [98].

The main mechanisms that appear to underlie resistance to immunotherapy in cancer patients are immunoediting and defects of the T cell compartment, both occurring with varying intensity in different patients, hence probably explaining the pronounced heterogeneity in the observed clinical responses [67,99,100].

Immunoediting is a naturally occurring process that takes place during tumor progression but also during immunotherapy and leads either to cancer suppression or promotion [67]. During immunoediting, the immunogenicity of tumor antigens could be reshaped, mostly after prolonged selective pressure, such that the immune system is driven to “immunological ignorance” and immunosurveillance mechanisms are no longer capable of recognizing and fighting tumor variants [101]. Evidently, the close proximity between malignant cells and bystander cells within the germinal centers in CLL provides the opportunity for longitudinal interplay that progressively shapes CLL cells’ immunogenicity [102]. Although an extensive description of immunoediting is beyond of the scope of this review, it should be noted that the identification of new immunogenic tumor-derived epitopes represents an active area of research towards the discovery of new therapeutic targets. Presently, the relevance of immunoediting for CLL remains unclear.

**Figure 1:**
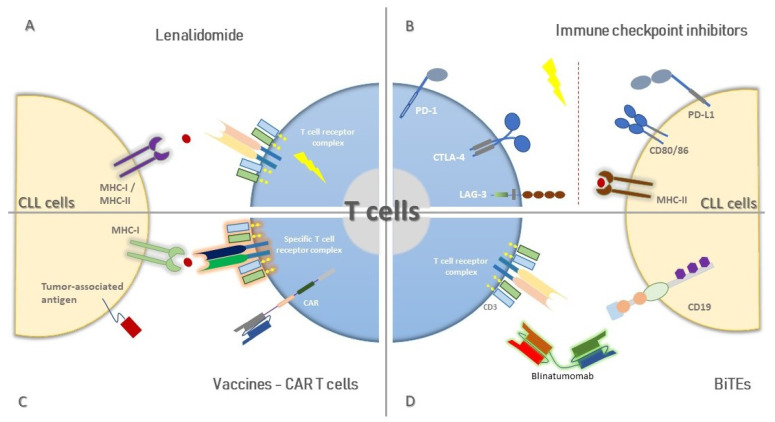
Mobilization of T cell functions through immunotherapy. The T cell compartment in CLL displays a great variety of defects leading progressively to immunosuppression; however, immunotherapy could restore T cell function through distinct ways. (**A**): Lenalidomide treatment increases T cell activation, amongst other effects; (**B**): IMiDs restrain inhibitory signaling mediated by overexpressed ligands and receptors in T cells and CLL cells, respectively; (**C**): Vaccine and CAR-T cell design based on the identification of TAAs could reinvigorate T-cell-mediated immunity; (**D**): BiTEs stimulate and drive cytotoxic T cell responses against specific molecules expressed by CLL cells.

Turning to the T cell compartment, this is severely dysfunctional in CLL with defects at the molecular and functional level that eventually foster CLL progression [103,104,105,106]. Nevertheless, there are also reports for T-cell-mediated leukemia control, particularly considering the identification of antigen-specific T cells that can mount anti-CLL responses in murine models [104,107]. Deciphering the aspects of this controversial implication of T cells in CLL pathophysiology is therefore of the utmost importance for refining the management of patients with CLL (Figure 1).

## 5. T Cell Compartment Defects as Targets for Immunotherapy

### 5.1. Imbalances in T Cell Subpopulations and Functions

Perhaps the most uniformly described T cell irregularity in CLL cohorts is the imbalance amongst different T cell subpopulations. More precisely, early findings documented elevated numbers of T cells in the periphery of CLL patients that mostly stem from CD8^+^ T cells. The impact of the resulting inversion in CD4^+^/CD8^+^ cell ratios remains a controversial issue, with some studies associating CD8^+^ cell increase with disease progression and shorter progression-free survival (PFS), thus contrasting others that correlated elevated numbers of CD8^+^ cells with an indolent clinical course [108,109]. However, the differentiation status, as well as the expression of co-stimulatory/co-inhibitory molecules on T cell surface, may be more clinically relevant than the relative proportion of the CD4^+^ versus the CD8^+^ cell lineage. Indeed, CD8^+^ T cells in CLL have been described as terminally differentiated towards an effector-memory phenotype, whereas they have lost their proliferation capacity, while also their cytotoxic function is severely compromised [110,111]. Along with the inability of CD8^+^ cells to control tumor growth due to reduced cytotoxicity, pro-tumoral signaling that supports CLL cells’ survival is also induced by the CD4^+^ T cell fraction in CLL [112,113].

On these grounds, for immunotherapeutic interventions that are based on T cell cytotoxicity, such as BiTEs and CAR-T cells, it would not be unreasonable to argue that their efficacy may be a priori limited. In that regard, it is relevant to mention that pre-clinical studies have demonstrated that blinatumomab treatment could lead to the reinvigoration of T cell anti-tumor responses ex vivo, through T cell activation, proliferation, cytokine expression and, finally, antigen-independent cytotoxicity from both CD4^+^ and CD8^+^ T cells [114]. Against that, however, the generation and sustained performance of specific anti-tumor T cell clones is known to be affected by several mechanisms, including the immunoediting process, the absence of naïve T cells, as well as the increase in immunosuppressing subpopulations, most of which are occurring in CLL as well [115].

The fine balance between the increased numbers of Tregs and the Th17 helper subpopulation in patients with CLL has prognostic value at early stages of the disease. Increased numbers of Tregs have been observed in CLL in comparison with healthy controls, resulting in immunosuppression, in contrast to Th17 cells that control the outgrowth of the former subpopulation [116,117,118,119].

In light of this evidence, it becomes urgent to delve deeper in the complex cellular interactions taking place in the CLL TME, similar to what has been attempted in other types of cancer, including both hematological and solid cancers. Along these lines, results from studies of blinatumomab in B-ALL suggest that removal of Tregs convert the non-responders to responders [120]. Moreover, in a murine model of lung cancer, stimulation of tumor-specific CD8+ cytotoxic cells with Th17 cells led them to unleash their cytotoxic function [121]. Finally, studies in melanoma have demonstrated durable responses after the adoptive transferring of Th17 cells, collectively providing a rationale for novel T-cell-based immunotherapies [122].

### 5.2. Defective Immune Synapse Formation

Although tumor-specific antigens are expressed by CLL cells (e.g., the ROR1 antigen) and are further effectively presented by MHC class I and class II molecules, cognate T cells fail to mount strong anti-tumor immune responses [123,124,125,126]. This tolerogenic behavior of T cells is partly due to differentially expressed genes that affect the formation of the cytoskeleton and vesicle trafficking, processes essential for T cell activation [127]. Studies have documented that these defects are imposed by CLL cells though direct contact, as they can also drive similar changes in T cells of healthy donors [128]. Further investigations have demonstrated that CLL cells escape immune cell recognition not only due to potential immunoediting and poor APC function, but also due to defects in immune synapse formation with the cognate T cells [129]. Along with the implication of impaired immunological synapse in the natural anti-tumor responses, this phenomenon is also relevant for immunotherapeutic approaches that rely on T cell–CLL cell interactions, including CAR-T cells and BiTEs. For both cases, restored immunological synapse formation has been observed after treatment with lenalidomide, proposing this regimen as a rational option for combination therapy [129,130,131].

### 5.3. Functional Incapacitation as an Aspect of Terminal Differentiation?

T cell exhaustion is established by persistent antigenic stimulation initially described in chronic viral infections and lately in different types of cancer and is characterized by T cells that have lost effector functions and display low proliferation capacity and defective cytotoxic responses [41]. Exhausted T cells display major alterations in their expression profiles that lead to reformed cytokine production and, most importantly, overexpression of multiple inhibitory ligands (e.g., PD-1, Lag-3, Tim-3) [132]. Studies in CLL have documented that both CD4^+^ and CD8^+^ T cells express the exhaustion markers CD244, CD160, as well as PD-1, the receptor for PD-L1 that is overexpressed by CLL cells [41,133]. Of note, BCRi treatment has been shown to reverse exhaustion, leading to an increase in the expression of activation markers in effector memory T cells and improved immune synapse formation [89].

In addition to exhaustion, senescence has been proposed as an alternative cellular mechanism that controls T cell proliferation [134]. Both exhaustion and senescence occur during the physiological aging process but, most importantly, during chronic viral infections, and are further associated with the dampening of immune responses in cancer patients [134,135]. Both exhausted and senescent T cells are characterized by the inability to exert rapid clonal expansion, and they are progressively leading to immunosuppression [136].

Exhaustion and senescence are extensively studied in cancer with a view to reinvigorating anti-tumor responses through targeting critical pathways. Evidence supports that it is much safer to target pathways implicated in exhaustion since senescence controls malignant transformation driven by DNA damage [135]. That said, ICIs represent an appealing treatment option, considering that they can partially halt T cell exhaustion leading to the restoration of T cell effector functions, as mentioned previously [40,44]. However, reinstation of the proliferative capacity and functionality remains an unmet need also when harnessing patients’ T cells for the generation of CAR T cells. In other contexts, but not yet CLL, efforts to reverse exhaustion and interrupt senescence in CAR T cells have been made; however, T cells displayed limited secretion of effector cytokines, at least for the latter approach [137]. Tackling this obstacle, designs of CAR T cells with the ability to secrete PD-1, CTLA-4 or PD-L1 antibodies have emerged and are currently in the phase of clinical trials [138].

## 6. Immunogenetic Cues Support the Existence of Specific Anti-CLL T Cell Responses

**Figure 2:**
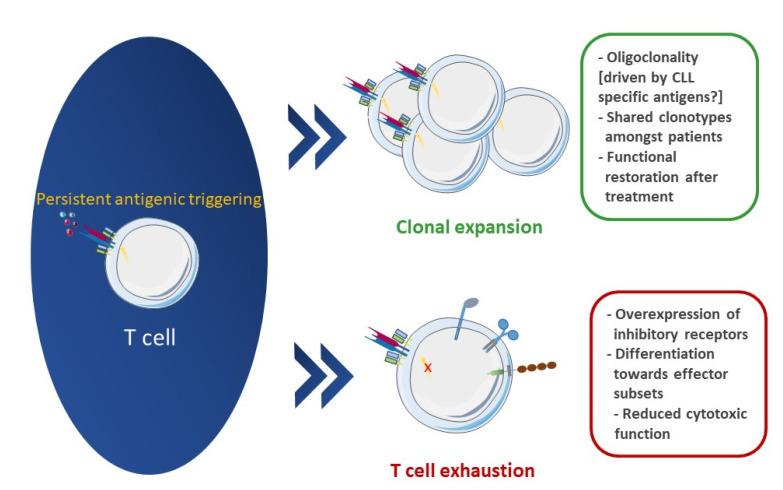
Chronic antigenic stimulation in CLL TME: functional and immunogenetic evidence. Persistent interactions with antigens lead T cells to functional exhaustion; however, clonal expansions as a response to these antigens occur in CLL patients.

The established states of T cell exhaustion and senescence in patients with CLL strongly allude to antigenic pressure. In addition to this functional evidence, immunophenotypic and immunogenetic evidence amply supported that antigen selection shapes the TR repertoire in CLL. In more detail, early immunophenotypic studies documented T cell clonal expansions in CLL [139]. More recently, in-depth characterization of the TR gene repertoire by next-generation sequencing (NGS) has disclosed T cell oligoclonality throughout the disease course that stems mainly from cytotoxic T cells [140,141]. Furthermore, the identification of patients bearing identical or highly similar TR clonotypes, not previously described in other entities, highlights the possibility that a fraction of T cells in CLL patients specifically recognize leukemia-associated antigens [89]. In line with this observation, longitudinal studies of the TR gene repertoire of patients with CLL under BCRi treatment revealed the expansion of pre-treatment clones over a deepening clinical response that in some cases was also accompanied by functional restoration [89] (Figure 2).

On these grounds, determining the precise selecting antigens for T cells in the CLL TME remains of great relevance in view of current T cell immunotherapeutic developments. In this direction, studies of HLA ligandome have documented immune recognition of disease-specific antigens arising from nonmutant CLL-derived peptides, highlighting novel targets for approaches aimed at overcoming immune ignorance, e.g., vaccination, BiTEs and CAR-T cell designs [142].

## 7. Concluding Remarks

A definitive cure in CLL still remains an unmet need. However, major advances in our understanding of this disease have translated in many and increasingly more effective therapeutic options. Realizing that CLL cells depend on extracellular cues has paved the way to new therapeutic modalities which target various TME components, thus depriving the malignant cells of important trophic triggers. Interestingly, despite the inherent T cell defects characterizing CLL, the immune system retains the potential to mount anti-tumor responses, at least to a degree. Therefore, the stimulation of immune responses through immunotherapy is a logical and promising approach for improving outcomes in patients with CLL; quite a lot has already been achieved, but much more remains to be done.
